# Clinical trial participant characteristics and saliva and DNA metrics

**DOI:** 10.1186/1471-2288-9-71

**Published:** 2009-10-29

**Authors:** Denise M Nishita, Lisa M Jack, Mary McElroy, Jennifer B McClure, Julie Richards, Gary E Swan, Andrew W Bergen

**Affiliations:** 1 Center for Health Sciences, SRI International, Menlo Park, CA, 94025, USA; 2 Group Health Center for Health Studies, Seattle, WA, 98101, USA

## Abstract

**Background:**

Clinical trial and epidemiological studies need high quality biospecimens from a representative sample of participants to investigate genetic influences on treatment response and disease. Obtaining blood biospecimens presents logistical and financial challenges. As a result, saliva biospecimen collection is becoming more frequent because of the ease of collection and lower cost. This article describes an assessment of saliva biospecimen samples collected through the mail, trial participant demographic and behavioral characteristics, and their association with saliva and DNA quantity and quality.

**Methods:**

Saliva biospecimens were collected using the Oragene^® ^DNA Self-Collection Kits from participants in a National Cancer Institute funded smoking cessation trial. Saliva biospecimens from 565 individuals were visually inspected for clarity prior to and after DNA extraction. DNA samples were then quantified by UV absorbance, PicoGreen^®^, and qPCR. Genotyping was performed on 11 SNPs using TaqMan^® ^SNP assays and two VNTR assays. Univariate, correlation, and analysis of variance analyses were conducted to observe the relationship between saliva sample and participant characteristics.

**Results:**

The biospecimen kit return rate was 58.5% among those invited to participate (n = 967) and 47.1% among all possible COMPASS participants (n = 1202). Significant gender differences were observed with males providing larger saliva volume (4.7 vs. 4.5 ml, p = 0.019), samples that were more likely to be judged as cloudy (39.5% vs. 24.9%, p < 0.001), and samples with greater DNA yield as measured by UV (190.0 vs. 138.5, p = 0.002), but reduced % human DNA content (73.2 vs. 77.6 p = 0.005) than females. Other participant characteristics (age, self-identified ethnicity, baseline cigarettes per day) were associated with saliva clarity. Saliva volume and saliva and DNA clarity were positively correlated with total DNA yield by all three quantification measurements (all r > 0.21, P < 0.001), but negatively correlated with % human DNA content (saliva volume r = -0.148 and all P < 0.010). Genotyping completion rate was not influenced by saliva or DNA clarity.

**Conclusion:**

Findings from this study show that demographic and behavioral characteristics of smoking cessation trial participants have significant associations with saliva and DNA metrics, but not with the performance of TaqMan^® ^SNP or VNTR genotyping assays.

**Trial registration:**

COMPASS; registered as NCT00301145 at clinicaltrials.gov.

## Background

Clinical trial and epidemiological studies need high quality biospecimens from a representative sample of participants to investigate genetic influences on treatment response and disease. DNA is typically extracted from one of several possible tissue sources including buccal cells, saliva, and blood, using a number of different methods [1]. Whole blood generally yields large amounts of high quality DNA but whole blood collection has limitations such as the logistics and expense of arranging for phlebotomy, lower response rates due to the invasiveness of the procedure, and time and temperature sensitive shipping and storage requirements [2]. In light of these limitations, clinical trials and epidemiological studies are increasingly using saliva as a source of human DNA because saliva can be non-invasively collected, sent through the mail, and stored at room temperature for years before extraction [3,4]. Response rates associated with salivary biospecimen collection have been shown to be higher than with whole blood [5]. Studies have shown that saliva collected from Oragene^® ^DNA Self-Collection Kits (DNA Genotek, Inc., Ottawa, Ontario, Canada), as well as other methods, yield high quality DNA, that can be used as an alternative to DNA extracted from blood [4-9].

One potential limitation associated with the use of saliva DNA is the potential effect of variable percentages of human and non-human DNA on various DNA quantification and genotyping methods. The most commonly used DNA quantification methods, such as ultraviolet spectrophotometric absorbance ("UV") and fluorescent dyes such as PicoGreen^® ^("PG") do not differentiate between human and non-human DNA, but quantitative real time Polymerase Chain Reaction ("qPCR") or hybridization methods using human specific oligonucleotide primers are human DNA specific [10-13]. In one study, an estimate of the fraction of human DNA in DNA extracted from saliva ranged from 11-100% [9]. Two studies have shown increased amounts of specific types of bacteria in saliva of smokers compared to that of nonsmokers [14,15]. A study evaluating the effects of the fraction of human DNA present in DNA extracted from saliva and buccal samples on genotyping using the Affymetrix GeneChip^® ^Mapping 500K Array suggested that samples containing < 30% human DNA had poor genotyping performance [16].

Because biospecimens are an essential component for clinical biomarker or genetic epidemiological studies, any demographic, behavioral, processing, or quantification factors that could impact the quality or suitability of biospecimens for molecular analysis should be investigated. The purpose of this report is to investigate associations of smoking cessation trial participant demographic and behavioral variables with saliva and saliva DNA characteristics, including saliva volume, saliva and DNA visual clarity, total DNA yield, and human DNA concentration, as well as the relationship between saliva characteristics and total and human DNA concentrations and genotyping performance.

## Methods

### Participants and saliva collection

All recruitment, consent, screening and data collection methods were reviewed and approved by the Institutional Review Boards of SRI International (SRI) and Group Health (GH). Participants were recruited from the Comprehensive Medication Program And Support Services (COMPASS) study, a randomized trial sponsored by the National Cancer Institute (NCT00301145) that recruited participants from members of Group Health (GH), a large health care system in Washington state. COMPASS was designed to compare the effectiveness of three versions of a smoking cessation behavioral therapy combined with Chantix^® ^(varenicline tartrate, Pfizer) [Swan GE, McClure JB, Jack LM, Zbikowski SM, Javitz HS, Catz SL, Deprey M, Richards J, McAfee TA: Behavioral Counseling and Varenicline Treatment for Smoking Cessation. American Journal of Preventive Medicine (submitted)]. COMPASS participants were invited by telephone to provide a saliva sample for a National Institute of Drug Abuse sponsored study being conducted by the Pharmacogenetics of Nicotine Addiction and Treatment (PNAT) consortium http://www.pharmgkb.org/contributors/pgrn/pnat_profile.jsp. Those who agreed to participate were mailed a consent form and an Oragene^® ^DNA Self-Collection Kit (disk format OG-250, DNA Genotek, Ottawa, Ontario, Canada). Participants were instructed to follow the manufacturer's instructions for saliva collection which were to 1) rinse the mouth with drinking water and wait at least five minutes before spitting saliva into the container; 2) spit enough saliva to reach an indicated level on the container; 3) screw the container cap on securely and then shake the container for at least 10 seconds. Samples were mailed back to the lab at room temperature, and participants were paid $25 upon sample receipt. If there was evidence of low yield or low DNA quality after DNA extraction, quantification and genotyping, participants were re-contacted and asked to provide a second sample. Additional written instructions to refrain from eating 30 minutes before saliva collection was added to the second mailed kits to help prevent any carryover of food particles.

### DNA extraction

Saliva was stored at room temperature for up to 7 months in the Oragene^® ^disks until DNA extraction. According to the manufacturer of the disks, saliva DNA is stable for over 2 years at room temperature [3]. Prior to DNA extraction, saliva samples were visually inspected and rated as "clear" or "cloudy" by the same laboratory analyst, as there were some samples with notable phlegm or cloudiness. DNA was extracted from saliva samples using the manufacturer's protocol for manual purification of DNA from 4.0 mL, PD-PR-015 Issue 2.0. The entire saliva sample was extracted with reagent volumes adjusted to maximize the amount of DNA recovered. Briefly, samples were mixed by inversion, and then incubated overnight at 50°C. Samples were transferred to a centrifuge tube and mixed with Oragene^® ^purifier, incubated on ice, then centrifuged at either 2500 × g or 3000 × g (protocol versions 1 or 2, respectively) for 20 minutes to pellet the denatured protein. The supernatant was transferred to a new tube and DNA was precipitated by adding an equal volume of 100% ethanol. The DNA pellet was washed with 70% ethanol, dried, and resuspended with DNA hydration solution (Qiagen, Valencia, CA). DNA was incubated at 50°C for 1 hour, followed by incubation at room temperature overnight to ensure complete rehydration. A high speed centrifugation step at 15,000 × g was performed to remove additional impurities. DNA samples were also rated as "clear" or "cloudy" by the same laboratory analyst via visual inspection of the tube when held up against a black background and compared to a referent tube that was previously noted as cloudy. DNA samples were stored at 4°C for up to 3 days until quantification.

### DNA quantification

DNA samples were quantified using three methods - UV, PG and qPCR. UV absorbance was measured via the Nanodrop Technologies NanoDrop^® ^ND-1000 Spectrophotometer (Wilmington, DE) and software. Genotyping was done on dilutions based on UV concentrations. A portion of the dilution tubes were rechecked via UV, and then subsequently quantified with two additional different quantification methods. For PG, double stranded DNA concentration was assessed using Quant-iT™ PicoGreen^® ^dsDNA Reagent (Invitrogen™, Carlsbad, CA) on a Tecan GENios™ (San Jose, CA) spectrophotometer. Human nuclear DNA concentration was assessed by an intra-*Alu*-based quantitative PCR (qPCR) assay in a singleplex PCR of 25 ul and otherwise as described [13]. Samples were amplified with a standard curve and three no-DNA template controls on each plate. The standard curve, performed in duplicate, ranged from 100 ng to 0.01 ng per well for each qPCR plate using lymphoblastoid cell line DNA NA07019 (Coriell Cell Repositories, Camden, New Jersey). qPCR reactions were analyzed using SDS v.1.2.3 software (Applied Biosystems), with manual baseline calling. DNA yield was estimated by measured concentration × dilution factor × the total resuspension volume. An estimate of the fraction of human DNA in saliva DNA was obtained by dividing the qPCR measurement by the PG measurement.

### Genotyping

Genotyping was performed on all DNA samples for 11 TaqMan^® ^single nucleotide polymorphism (SNP) assays (Applied Biosystems) and for two variable number of tandem repeat (VNTR) polymorphisms. Positive controls for each TaqMan^® ^cluster group and no DNA template controls were included on each plate using the suggested protocol [17]. To evaluate discordance, 10% of the samples were genotyped in duplicate, as was the second saliva DNA sample collected for 18 participants. TaqMan^® ^assays were analyzed using SDS v1.2.3 software. Other genotyping assays were performed for VNTRs in the 3'UTR of SLC6A3 [18] and in Exon 3 of DRD4 [19].

### Statistical analysis

Univariate, correlation and analysis of variance analyses were performed using SAS version 9.1 (Cary, NC). Tests of statistical significance used a Type I error rate of 0.05.

## Results

### Saliva sample collection and participant demographic and behavioral characteristics

**Figure 1:**
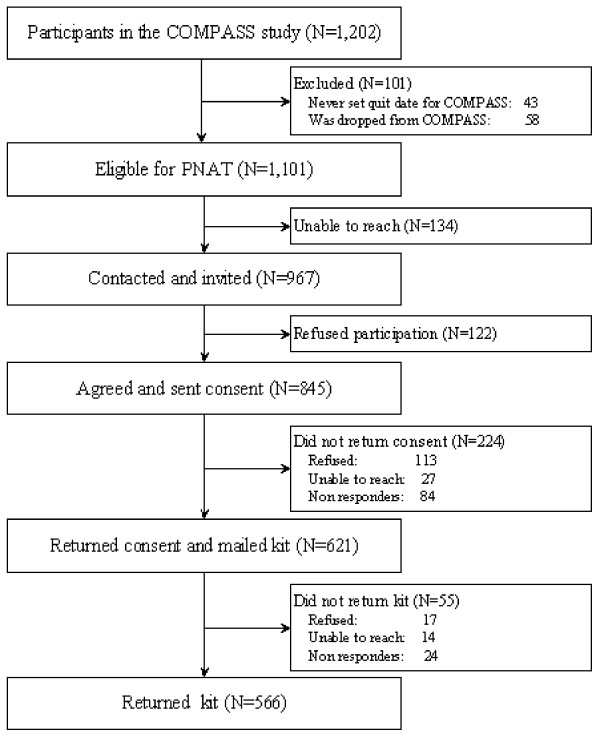
**Saliva biospecimen collection from COMPASS participants**.

Of the total of 1,202 participants randomized to treatment in the COMPASS study [Swan GE, McClure JB, Jack LM, Zbikowski SM, Javitz HS, Catz SL, Deprey M, Richards J, McAfee TA: Behavioral Counseling and Varenicline Treatment for Smoking Cessation. American Journal of Preventive Medicine (submitted)], a total of 1,101 were eligible for this PNAT study (see Figure 1). Successful contact was made with 967 participants (87.8% of those eligible) and a total of 566 participants (58.5% of those contacted and 51.4% of those eligible) returned saliva kits. One kit received was empty, resulting in usable saliva samples from 565 participants. The overall participant characteristics can be seen in Table 1. There were no significant differences between the N = 566 COMPASS participants who returned a saliva collection kit (47.1%) and the N = 636 (1202-566) other participants with respect to gender, ethnicity (self identified non-Hispanic Caucasian), years of formal schooling, and amount smoked. Those who did return a saliva collection kit were significantly older than those who did not (48.1 vs. 45.0 years, t = -5.2, p < .0001).

**Table 1:** COMPASS participant and sample characteristics

Characteristic	M (SD) or %	Range
*Participants (n = 566)*		

Age (years)	49.1 (11.4)	19.0-76.8
Gender (% female)	67.8	
Ethnicity (% NHC)	88.5	
Years of formal schooling	14.2 (2.2)	6.0-24.0
Cigarettes per day (CPD)	20.1 (8.2)	4.0-80.0

*Saliva (n = 565)*		

Time between kit receipt and DNA extraction (days)	63.8 (43.1)	2.0-203.0
Volume (ml)	4.5 (1.1)	1.0-7.5^†^
Visual clarity (% "cloudy")	29.6	

*DNA (n = 539)*		

Processed with protocol 2 (%)	80.0	
DNA yield by UV (ug)	155.2 (156.1)	1.5-1153.3
DNA yield by PG (ug)	82.8 (79.4)	0.9-549.3
DNA yield by qPCR (ug)	61.5 (58.6)	0.8-344.3
Visual clarity (% "cloudy")	17.2	
A260/280	1.83 (0.10)	1.42-2.13

^†^An 8.5 ml sample was also included that was derived from two saliva kits extracted together. The range shown in Table 1 show saliva volumes from single saliva collection kits.

After DNA extraction, quantification and genotyping, 38 participants were recontacted to request a second saliva sample due to evidence of low yield (n = 15) or low initial genotyping rate (n = 23) when 3 or more out of 7 initial TaqMan^® ^assays tested had failed. Half of the attempted requests for a second saliva sample (n = 19) resulted in a kit being returned, one of which was empty. There were no significant differences in age, gender, ethnicity, years of formal schooling or amount smoked between those who provided two samples compared to those who provided one. In all cases but two the second sample appeared superior with respect to genotyping completion rate and was used instead of the first sample (i.e., only one sample was used per person), resulting in a total of 565 samples for analysis. In addition, for the 7 usable samples that had a second sample returned because of an initial low yield, 6 gave similar low yields (data not shown).

### Saliva sample and saliva DNA characteristics

**Figure 2:**
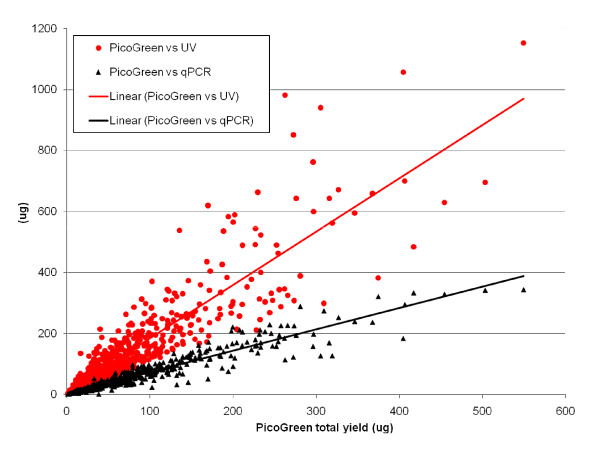
**Total DNA yield measured by PicoGreen^® ^vs human DNA yield measured by qPCR, and vs total DNA yield measured by UV, N = 539 COMPASS saliva DNA samples**.

**Figure 3:**
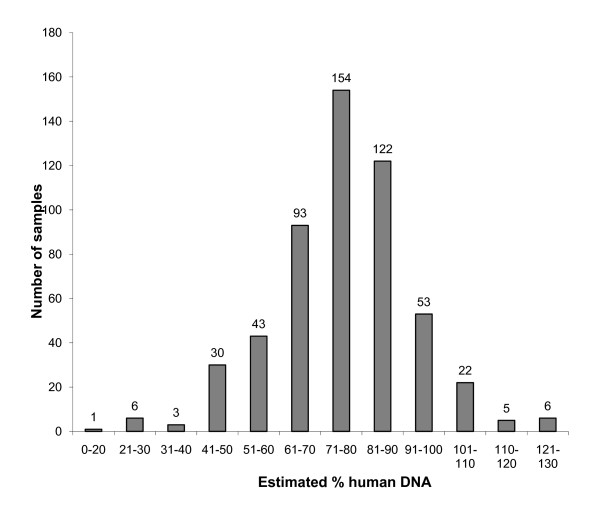
**% human DNA for N = 538 COMPASS saliva DNA samples**.

The overall saliva and saliva DNA characteristics can be seen in Table 1. DNA analysis (N = 539) was limited to one sample per participant for which all three quantification measurements were available. Total DNA yield measurements were all highly significantly correlated (Spearman) (UV vs PG (r = 0.922, P < 0.001), UV vs qPCR (r = 0.832, P < 0.001), and PG vs qPCR (r = 0.962, P < 0.001)) (Figure 2) and saliva volume was significantly correlated with the total DNA yield as measured by each of the three methods using UV, PG, and qPCR (r = 0.33, 0.28, 0.25 respectively, all P < 0.001), but inversely correlated with the calculated % human DNA (r = -0.148, P = 0.005). A median of 77%, a mean (SD) of 76.1% (17.1%), and a range of 2.4-159% estimated human DNA content was obtained (Figure 3). The single sample at 159% was excluded from the figure, but not from analysis. For the sample concentrations for all three quantification methods see Additional file 1.

### Participant and saliva or saliva DNA associations

When the time between kit receipt and extraction is split at the median (≤ 55 days = "Early Extraction" and ≥ 56 days = "Later Extraction"), there were no significant differences for the three DNA yield measurements or for genotyping completion rate (data not shown). The same was true when a linear model was applied (data not shown). The mean (SD) A260/280 for the earlier extractions vs. later extractions was 1.82 (0.10) and 1.86 (0.11), t_(537) _= -4.28, P < 0.001 respectively, and although significantly different, both were in an acceptable range. When a linear model was applied, the A260/A280 remained significant (r = 0.24, P < 0.01).

There were statistically significant associations between participant demographic and behavioral characteristics and saliva and DNA volume, clarity, and yield (Tables 2 and 3). Females provided significantly less saliva and had significantly lower total DNA yield by UV but significantly higher % human DNA yields, and provided saliva which was clear significantly more often than did males. Non-Hispanic Caucasians provided significantly more saliva, which was cloudy significantly more often and which resulted in significantly more total DNA yield by UV, than all others. Age was significantly associated with reduced saliva clarity and reduced % human DNA yield. CPD were significantly associated with increased saliva volume and with saliva that was clear significantly less often.

**Table 2:** Saliva and saliva DNA quantity by COMPASS participant characteristics

	Saliva Volume	DNA Yield UV	% Human DNA
	N = 565 samples	N = 539 samples	N = 539 samples
*Overall mean (SD)*	4.5 (1.1)	155.2 (156.1)	76.1 (17.1)

*Correlations*			

Age			
R value	0.02	0.05	-0.10
P value	0.684	0.270	0.027

CPD*			
R value	0.10	0.03	-0.02
P value	0.016	0.448	0.719

*Average by category*			

Gender			
Female	4.5 (1.1)	138.5 (132.4)	77.6 (16.8)
Male	4.7 (1.1)	190.0 (192.3)	73.2 (17.2)
P value	0.019	0.002	0.005

Ethnicity			
Other	4.3 (1.0)	122.0 (128.5)	76.7 (18.8)
NHC^†^	4.6 (1.1)	159.3 (159.0)	76.0 (16.8)
P value	0.042	0.040	0.758

*CPD = cigarettes per day. ^†^NHC = non-Hispanic Caucasian.

**Table 3:** Saliva and DNA clarity by COMPASS participant characteristics

	Saliva Clarity (N = 533 samples)			DNA Clarity (N = 539 samples)		
	
	Clear	Cloudy	P-value	Clear	Cloudy	P-value
Age, mean (SD), y	48.1 (11.4)	51.1 (11.0)	0.005	48.9 (11.4)	49.4 (11.7)	0.733

CPD, mean (SD)	19.0 (7.0)	22.2 (9.1)	<0.001	19.7 (7.4)	21.2 (9.4)	0.143

Gender						
Female	75.1%	24.9%	<0.001	84.9%	15.1%	0.057
Male	60.5%	39.5%		78.3%	21.7%	

Ethnicity						
Other	85.0%	15.0%	0.008	87.1%	12.9%	0.351
NHC	68.4%	31.6%		82.4%	17.6%	

Among saliva and saliva DNA samples judged to be cloudy (Table 4), saliva volumes were significantly increased and DNA yields were increased to approximately twice that of clear samples, while estimated % human DNA was reduced to approximately 92% of clear samples. DNA purity (A260/280 ratio) and genotyping completion rate were not significantly associated with saliva or saliva DNA sample clarity. The DNA extraction protocol version that used higher centrifuge speeds showed no significant differences in DNA yield, DNA clarity, or genotyping success rate (data not shown). The genotyping completion rate for each SNP assay tested was over 98% per subject and for the *SLC6A3 *and *DRD4 *VNTRs were 98.5% and 97.5% per subject. All genotype distributions were in Hardy Weinberg Equilibrium and no genotype discordance was found when re-genotyping 10% of the samples or when comparing called genotypes from the initial saliva sample to the second saliva sample collected from 18 participants. Out of the 14 second saliva samples received from individuals recontacted because of poor genotyping completion rates, 12 had improved genotyping rates and 2 did not. The mass of human DNA added to genotyping reactions was not associated with TaqMan^® ^genotype completion rate (Pearson r = 0.064, P = 0.122), but was significantly associated with VNTR genotype completion rate (Pearson r = 0.173, P < 0.001).

**Table 4:** Saliva and saliva DNA yield, purity and genotyping rate by sample clarity, COMPASS participants

	Saliva			DNA		
	
Sample variable	Clear	Cloudy	P*	Clear	Cloudy	P*
N samples	375	158		446	93	

Saliva volume mean (SD), ml						
	4.5 (1.1)	4.7 (1.1)	0.037	4.4 (1.0)	5.2 (1.0)	<0.001

*DNA based measurements mean (SD)*						
UV, mean (SD), ug	121.7 (111.5)	236.4 (210.5)	<0.001	120.2 (104.2)	323.0 (235.8)	<0.001
PG, mean (SD), ug	69.3 (62.9)	115.6 (102.6)	<0.001	64.2 (55.5)	171.8 (111.0)	<0.001
qPCR, mean (SD), ug	54.1 (51.2)	79.4 (70.1)	<0.001	49.1 (44.5)	121.4 (78.3)	<0.001
A260/280	1.84 (0.10)	1.84 (0.12)	0.746	1.84 (0.11)	1.83 (0.11)	0.617
% human DNA	78.0 (16.4)	71.3 (17.6)	<0.001	77.0 (16.9)	72.0 (17.4)	0.010
Genotype (%)	98.5 (9.6)	98.7 (7.4)	0.757	98.4 (9.7)	99.4 (4.0)	0.104

*t test P

## Discussion

In this study we observed significant influences of demographic and behavioral characteristics on saliva and DNA quantity and quality as well as significant associations among saliva biospecimen and DNA characteristics. Gender and ethnicity exhibited associations with saliva volume and DNA quantities, similar to a study that collected buccal cells by a mouthwash method where males had greater buccal DNA yields (quantified by UV) than females [20]. A recent study suggested a relationship between genetic relatedness of participants and the quality of the DNA prepared from their saliva [12], although is unclear if this is from potentially heritable biological or environmental factors that inhibit PCR.

Saliva volumes were significantly inversely associated with saliva biospecimen clarity and saliva DNA clarity. Although saliva and saliva DNA biospecimen samples judged to be "cloudy" tended to have increased total DNA yield and decreased % human DNA, they had similar genotyping success rates and A260/280 ratios as "clear" samples. The timing of DNA extraction was not related to total DNA yield or genotyping performance, which is consistent with DNA Genotek's report that the saliva samples are stable at room temperature and yield high quality DNA [3].

The estimated human DNA content of DNA extracted from COMPASS saliva samples (median, 77%) is similar to previously reported results for DNA extracted from saliva biospecimens collected using Oragene^® ^kits, where median human DNA content has been reported to be 68% and 80% using prothrombin and RNaseP qPCR assays, respectively [9,21] and is higher than that reported for other oral-cavity related biospecimen samples such as cytobrush (11.5%) and mouthwash samples (49.5%) [10]. This may be due to the antibacterial reagents in the Oragene kits that prevent bacterial growth. We obtained some values greater than 100% for estimated % human DNA content, which may be explained by our use of a ratio of two different quantification methods, each with substantial variance.

The genotyping completion rate for all SNP and VNTR genotyping assays was over 97%. These genotyping rates are similar to or higher than previously reported genotyping completion rates for DNA extracted from saliva samples using the same saliva collection kit [12,22]. In addition, the genotyping completion rate was substantially higher for our samples collected using the Oragene kit (98.5%) vs. a mailed buccal swab method (68%) for the same *SLC6A3 *VNTR polymorphism [18]. Previous reports have suggested some relationship with genotyping success and the amount of human DNA [12,16,22]. Although we did not observe a significant association between % human DNA and TaqMan^® ^SNP assay performance, more studies should be done to assess this relationship with other high throughput genotyping or sequencing platforms.

### Limitations

Saliva and DNA clarity observations did not use previously established validated protocols. For this study, although all clarity determinations were made by a single observer, there was no confirmation by a second observer. A limitation to the human DNA quantification method using a qPCR assay is that it may be sensitive to unknown PCR inhibitors present in greater concentration in the cloudy samples. Participants who had more total DNA as measured by PG also had more human DNA as measured by qPCR, even though the percent of human DNA decreased. As DNA yields from most samples with the recommended saliva volumes are adequate for genotyping, encouraging compliance with the saliva protocol to collect the recommended amount of saliva, and no more, is recommended.

Studies collecting biospecimen samples through the mail have variable return rates, and low biospecimen return rates could be a limitation. A study utilizing a different saliva collection procedure through the mail had an 80% return rate recruiting from a smoking cessation website [23], while another study using a mouthwash protocol through the mail had a 37% return rate from a cohort of smokers selected from participants of a smoking cessation intervention [24]. A study collecting cigarette smoking survey data and genetic material via buccal cell sampling through the mail had 25% of their total interviewees, representing 45% of those who agreed to receive a buccal cell collection kit, actually return their buccal cell kits [25]. With these reported percentages only representing a subset of the whole study, it is important to show that the biospecimens being analyzed were obtained from a representative sample of the whole study, or to find ways to improve participation in genetic aspects of population or clinically based studies. Having genetic material from a subset of all participants in a study could be a limitation and could potentially bias genetic association results. In our study, those who returned a kit were significantly older than those who did not, and this difference in age should be acknowledged when generalizing genetic association results to the entire COMPASS sample. Other limitations to collecting saliva samples in the mail include the inability to confirm compliance with collection protocols and to confirm that the biospecimen donor is the trial participant.

With numerous methods to quantify DNA, human specific DNA quantification may be the most useful for specific biospecimen types such as saliva and buccal swabs that can contain other non-human or biological contaminants. Although saliva and saliva DNA clarity did not seem to affect the observed genotyping call rate in this study, it is unclear to what degree these variables may affect different quantification methods which may affect input DNA mass and genotyping performance for other genotyping technologies.

## Conclusion

Findings from this study show that demographic and behavioral characteristics of smoking cessation trial participants have significant associations with saliva and DNA metrics. Potential saliva donors should be encouraged to provide the recommended amount of saliva, but no more than the recommended amount. Although some participant characteristics are associated with DNA quantity and clarity, the saliva collection process yields an amount of DNA sufficient for genotyping in most samples using TaqMan^® ^SNP and VNTR genotyping assays. Saliva samples collected through the mail can provide high quality DNA for genotyping and allow for easier biospecimen collection which can possibly increase study participation.

## Competing interests

The authors declare that they have no competing interests.

## Authors' contributions

DMN was responsible for manuscript writing and all laboratory procedures including biospecimen processing, quality control, genotyping, and data analysis. LMJ participated in the design and coordination of the COMPASS study, coordinated data management, performed statistical analysis, and contributed to manuscript writing. MM performed data management, data quality control, development of protocols for biospecimen collection, and oversaw the recruitment and biospecimen collection at SRI International. JBM contributed to the manuscript, participated in the design of the COMPASS study, and supervised all COMPASS and PNAT research activities at Group Health. JR coordinated the IRB approvals at GH, performed data management, and assisted in the development of protocols for biospecimen collection. GES contributed to the manuscript, conceived of and participated in the design and coordinating of the COMPASS study. AWB participated in the conception and design of the study, analytical strategies and manuscript writing. All authors read and approved the final manuscript.

## Pre-publication history

The pre-publication history for this paper can be accessed here:

http://www.biomedcentral.com/1471-2288/9/71/prepub

## Supplementary Material

Additional file 1 **DNA concentration (ng/ul) by three methods for N = 539 COMPASS saliva DNA samples**. The table shows the DNA concentrations measured by UV, PicoGreen, and qPCR for N = 539 samples. Click here for file
