# Supplementary material for: Clinical trial participant characteristics and saliva and DNA metrics
Source: BMC Med Res Methodol. 2009 Oct 29;9:71. doi: 10.1186/1471-2288-9-71 (PMC2776600; doi:10.1186/1471-2288-9-71)
Supplement: Additional file 1 — DNA concentration (ng/ul) by three methods for N = 539 COMPASS saliva DNA samples. The table shows the DNA concentrations measured by UV, PicoGreen, and qPCR for N = 539 samples. [file 1471-2288-9-71-S1.doc]

**Additional file 1. DNA concentrations (ng/ul) by various methods for N=539 samples.**

| **Sample ID** | **UV** | **PicoGreen** | **qPCR HUMAN** |
| --- | --- | --- | --- |
| 17390 | 154.6 | 69.4 | 56.2 |
| 17391 | 156.3 | 53.4 | 30.4 |
| 17392 | 158.3 | 63.8 | 49.7 |
| 17393 | 152.6 | 54.9 | 29.9 |
| 17394 | 142.9 | 62.5 | 58.1 |
| 17395 | 153.5 | 113.7 | 119.6 |
| 17396 | 179.2 | 134.3 | 126.2 |
| 17397 | 156.5 | 64.4 | 48.4 |
| 17398 | 153.5 | 68.0 | 43.6 |
| 17399 | 160.8 | 111.6 | 119.8 |
| 17400 | 153.3 | 110.3 | 81.1 |
| 17401 | 161.2 | 56.4 | 54.9 |
| 17402 | 154.8 | 61.5 | 51.5 |
| 17403 | 144.1 | 84.2 | 77.3 |
| 17404 | 198.2 | 165.6 | 146.7 |
| 17405 | 150.4 | 106.8 | 121.8 |
| 17406 | 154.8 | 86.4 | 75.6 |
| 17407 | 158.3 | 65.7 | 69.4 |
| 17408 | 163.1 | 80.6 | 53.9 |
| 17409 | 165.9 | 72.2 | 57.1 |
| 17410 | 147.0 | 83.3 | 73.4 |
| 17411 | 148.1 | 71.9 | 65.8 |
| 17412 | 155.0 | 36.0 | 22.9 |
| 17413 | 169.0 | 80.5 | 50.5 |
| 17417 | 152.1 | 71.5 | 82.1 |
| 17418 | 162.5 | 72.4 | 52.9 |
| 17419 | 158.6 | 50.2 | 27.1 |
| 17420 | 152.4 | 138.7 | 140.1 |
| 17421 | 142.2 | 113.4 | 113.3 |
| 17422 | 172.6 | 54.9 | 44.9 |
| 17423 | 151.3 | 93.6 | 97.6 |
| 17424 | 154.0 | 97.8 | 79.4 |
| 17425 | 173.5 | 148.2 | 137.8 |
| 17426 | 187.3 | 94.3 | 117.3 |
| 17427 | 194.4 | 51.9 | 22.5 |
| 17428 | 136.7 | 47.8 | 28.2 |
| 17429 | 159.2 | 74.7 | 51.7 |
| 17430 | 156.2 | 44.1 | 24.1 |
| 17431 | 150.9 | 73.4 | 73.1 |
| 17432 | 149.9 | 73.0 | 59.2 |
| 17433 | 148.4 | 134.9 | 132.4 |
| 17434 | 54.3 | 29.7 | 30.5 |
| 17435 | 151.9 | 61.7 | 51.1 |
| 17436 | 147.9 | 126.1 | 139.2 |
| 17437 | 148.9 | 114.1 | 98.0 |
| 17438 | 151.6 | 69.0 | 59.6 |
| 17439 | 156.5 | 68.7 | 58.8 |
| 17440 | 153.7 | 136.2 | 137.2 |
| 17441 | 114.0 | 76.2 | 69.7 |
| 17442 | 153.3 | 48.9 | 25.0 |
| 17443 | 149.3 | 128.8 | 158.2 |
| 17444 | 152.5 | 60.2 | 43.5 |
| 17445 | 150.3 | 68.9 | 48.2 |
| 17446 | 153.9 | 84.7 | 70.4 |
| 17447 | 153.3 | 86.2 | 71.0 |
| 17448 | 160.6 | 69.3 | 67.4 |
| 17450 | 148.8 | 53.9 | 28.7 |
| 17451 | 155.0 | 60.4 | 36.2 |
| 17452 | 154.2 | 54.1 | 40.2 |
| 17453 | 159.0 | 61.4 | 35.2 |
| 17455 | 153.3 | 54.2 | 36.2 |
| 17456 | 156.0 | 67.6 | 40.0 |
| 17457 | 152.2 | 159.4 | 147.1 |
| 17458 | 158.9 | 118.9 | 108.7 |
| 17459 | 156.5 | 81.9 | 65.4 |
| 17460 | 161.4 | 55.3 | 32.8 |
| 17461 | 159.0 | 52.4 | 25.9 |
| 17462 | 151.6 | 110.4 | 96.1 |
| 17463 | 152.6 | 71.5 | 57.9 |
| 17464 | 169.7 | 78.2 | 54.7 |
| 17465 | 152.4 | 72.3 | 70.5 |
| 17466 | 150.3 | 64.2 | 46.5 |
| 17467 | 147.3 | 36.4 | 15.2 |
| 17468 | 155.3 | 60.2 | 73.0 |
| 17469 | 169.5 | 93.5 | 82.6 |
| 17470 | 151.2 | 102.7 | 100.0 |
| 17471 | 136.1 | 33.4 | 0.8 |
| 17472 | 152.5 | 76.4 | 62.9 |
| 17473 | 148.0 | 97.6 | 75.0 |
| 17474 | 159.8 | 66.6 | 53.0 |
| 17475 | 160.7 | 35.8 | 23.1 |
| 17476 | 149.0 | 83.5 | 78.8 |
| 17477 | 151.6 | 67.5 | 45.9 |
| 17478 | 155.3 | 77.0 | 54.8 |
| 17479 | 152.0 | 127.8 | 96.8 |
| 17480 | 143.1 | 98.5 | 87.8 |
| 17481 | 141.6 | 116.0 | 100.3 |
| 17482 | 151.6 | NA | 27.8 |
| 17483 | 155.5 | 53.3 | 25.9 |
| 17484 | 157.6 | 67.1 | 45.7 |
| 17485 | 141.5 | 103.6 | 87.8 |
| 17486 | 149.3 | 56.0 | 38.0 |
| 17487 | 159.2 | 121.5 | 88.3 |
| 17488 | 152.3 | 49.8 | 26.5 |
| 17490 | 141.0 | 47.9 | 29.6 |
| 17491 | 175.7 | 115.3 | 104.1 |
| 17492 | 153.2 | 66.3 | 46.8 |
| 17493 | 160.5 | 41.4 | 24.4 |
| 17494 | 185.9 | 100.9 | 87.7 |
| 17495 | 187.8 | 110.7 | 90.8 |
| 17496 | 147.8 | 69.6 | 54.5 |
| 17497 | 143.5 | 96.9 | 80.6 |
| 17498 | 151.6 | 54.7 | 34.9 |
| 17499 | 155.8 | 114.4 | 93.7 |
| 17500 | 151.4 | 91.2 | 77.7 |
| 17502 | 148.5 | 102.6 | 95.0 |
| 17503 | 151.4 | 74.5 | 57.2 |
| 17504 | 156.6 | 32.6 | 15.1 |
| 17505 | 153.6 | 46.4 | 12.4 |
| 17506 | 138.1 | 103.1 | 77.8 |
| 17507 | 143.1 | 126.7 | 104.7 |
| 17508 | 146.2 | 108.1 | 85.1 |
| 17509 | 150.4 | 59.1 | 32.2 |
| 17510 | 158.8 | 133.0 | 104.6 |
| 17512 | 151.9 | 143.5 | 131.6 |
| 17513 | 148.5 | 119.9 | 101.3 |
| 17515 | 129.3 | 51.5 | 23.6 |
| 17516 | 156.6 | 68.3 | 46.2 |
| 17517 | 142.5 | 76.4 | 60.4 |
| 17519 | 147.9 | 75.7 | 53.3 |
| 17520 | 150.1 | 68.7 | 53.4 |
| 17521 | 182.7 | 132.2 | 113.9 |
| 17522 | 152.7 | 127.4 | 111.4 |
| 17523 | 169.5 | 122.3 | 88.6 |
| 17524 | 146.3 | 86.3 | 71.9 |
| 17525 | 159.7 | 165.0 | 146.6 |
| 17526 | 161.6 | 122.3 | 105.1 |
| 17528 | 204.6 | 70.8 | 42.2 |
| 17529 | 148.9 | 54.3 | 28.4 |
| 17530 | 150.2 | 113.6 | 106.3 |
| 17531 | 154.0 | 118.6 | 88.0 |
| 17532 | 154.0 | 81.8 | 50.2 |
| 17533 | 155.4 | 57.4 | 33.1 |
| 17534 | 144.0 | 103.9 | 79.2 |
| 17535 | 161.1 | 70.1 | 29.7 |
| 17536 | 146.9 | 96.3 | 85.1 |
| 17538 | 154.6 | 112.4 | 98.6 |
| 17539 | 153.6 | 95.5 | 68.4 |
| 17540 | 163.4 | 164.1 | 156.2 |
| 17541 | 151.6 | 81.4 | 60.7 |
| 17542 | 156.0 | 117.8 | 92.5 |
| 17543 | 154.6 | 99.4 | 68.7 |
| 17544 | 150.6 | 63.4 | 30.8 |
| 17546 | 173.0 | 92.0 | 71.5 |
| 17547 | 149.3 | 115.9 | 79.3 |
| 17548 | 144.3 | 89.2 | 88.3 |
| 17550 | 148.8 | 60.7 | 37.4 |
| 17551 | 158.8 | 51.1 | 39.5 |
| 17552 | 166.7 | 157.2 | 133.7 |
| 17554 | 148.2 | 49.3 | 20.1 |
| 17556 | 153.8 | 41.4 | 18.1 |
| 17557 | 154.2 | 76.0 | 62.6 |
| 17558 | 148.7 | 73.4 | 65.3 |
| 17559 | 142.1 | 87.5 | 90.4 |
| 17560 | 150.6 | 146.4 | 125.2 |
| 17561 | 154.5 | 100.9 | 89.5 |
| 17562 | 160.9 | 94.4 | 62.7 |
| 17564 | 152.0 | 106.7 | 130.2 |
| 17565 | 152.7 | 80.8 | 55.7 |
| 17566 | 148.9 | 106.8 | 111.1 |
| 17567 | 68.1 | 45.6 | 42.6 |
| 17568 | 160.3 | 73.2 | 32.9 |
| 17569 | 157.6 | 82.4 | 68.1 |
| 17570 | 149.8 | 75.3 | 61.2 |
| 17571 | 144.5 | 155.3 | 110.2 |
| 17572 | 148.9 | 35.1 | 9.7 |
| 17574 | 153.1 | 110.3 | 113.6 |
| 17575 | 165.8 | 115.3 | 107.3 |
| 17576 | 149.1 | 110.1 | 100.6 |
| 17577 | 174.7 | 150.4 | 120.4 |
| 17578 | 151.9 | 100.8 | 94.0 |
| 17579 | 153.0 | 96.2 | 62.3 |
| 17580 | 148.3 | 127.1 | 115.9 |
| 17581 | 157.7 | 79.7 | 65.7 |
| 17582 | 154.8 | 69.4 | 52.7 |
| 17583 | 151.0 | 84.9 | 71.6 |
| 17584 | 155.0 | 67.4 | 48.0 |
| 17585 | 161.1 | 121.6 | 85.8 |
| 17586 | 101.0 | 68.2 | 52.2 |
| 17587 | 150.8 | 56.1 | 27.5 |
| 17588 | 146.3 | 114.7 | 121.8 |
| 17589 | 155.6 | 59.3 | 45.2 |
| 17590 | 151.4 | 93.3 | 79.6 |
| 17591 | 157.5 | 97.4 | 82.2 |
| 17592 | 238.8 | 91.3 | 41.5 |
| 17593 | 185.4 | 66.1 | 48.0 |
| 17594 | 180.0 | 100.4 | 64.8 |
| 17596 | 151.4 | 121.3 | 77.8 |
| 17597 | 152.4 | 104.8 | 82.7 |
| 17598 | 177.1 | 75.4 | 46.2 |
| 17599 | 151.3 | 75.9 | 52.4 |
| 17600 | 154.0 | 91.4 | 73.0 |
| 17601 | 153.1 | 66.6 | 59.9 |
| 17602 | 173.8 | 81.5 | 52.6 |
| 17603 | 146.7 | 69.7 | 54.1 |
| 17604 | 158.3 | 109.2 | 69.0 |
| 17605 | 173.4 | 100.9 | 67.3 |
| 17606 | 148.9 | 95.5 | 90.7 |
| 17607 | 157.4 | 76.5 | 59.2 |
| 17608 | 150.5 | 123.9 | 104.0 |
| 17609 | 160.2 | 69.2 | 47.6 |
| 17610 | 116.2 | 80.8 | 59.4 |
| 17611 | 167.2 | 46.1 | 10.6 |
| 17613 | 151.9 | 66.4 | 49.5 |
| 17614 | 161.5 | 114.2 | 102.1 |
| 17615 | 136.4 | 76.7 | 59.1 |
| 17616 | 75.1 | 18.0 | 15.2 |
| 17617 | 145.4 | 51.7 | 39.5 |
| 17618 | 147.4 | 69.1 | 44.6 |
| 17619 | 149.8 | 111.1 | 84.6 |
| 17620 | 150.2 | 107.4 | 93.0 |
| 17621 | 153.7 | 112.7 | 77.8 |
| 17622 | 154.8 | 66.3 | 51.2 |
| 17623 | 147.4 | 107.7 | 103.1 |
| 17624 | 183.8 | 72.4 | 70.1 |
| 17625 | 113.1 | 84.8 | 78.2 |
| 17626 | 143.7 | 133.1 | 112.7 |
| 17627 | 142.9 | 109.7 | 84.7 |
| 17628 | 237.6 | 115.7 | 99.9 |
| 17629 | 141.2 | 68.5 | 42.3 |
| 17630 | 151.3 | 136.5 | 108.8 |
| 17631 | 147.9 | 112.1 | 94.9 |
| 17632 | 150.2 | 80.9 | 63.8 |
| 17633 | 154.5 | 54.1 | 28.5 |
| 17635 | 156.9 | 77.2 | 56.4 |
| 17636 | 172.5 | 103.3 | 102.9 |
| 17637 | 150.7 | 66.0 | 41.4 |
| 17638 | 227.3 | 164.8 | 138.4 |
| 17639 | 142.3 | 106.0 | 91.6 |
| 17640 | 151.1 | 171.1 | 144.6 |
| 17641 | 201.6 | 104.1 | 68.2 |
| 17642 | 106.0 | 83.2 | 72.1 |
| 17643 | 152.2 | 102.3 | 81.3 |
| 17645 | 155.8 | 62.9 | 54.5 |
| 17646 | 147.6 | 69.0 | 58.7 |
| 17648 | 161.1 | 73.0 | 53.4 |
| 17649 | 168.5 | 45.3 | 35.1 |
| 17650 | 148.9 | 61.0 | 41.8 |
| 17653 | 142.6 | 83.0 | 68.9 |
| 17654 | 156.9 | 88.3 | 70.5 |
| 17657 | 162.0 | 122.7 | 101.8 |
| 17658 | 169.3 | 77.2 | 45.1 |
| 17659 | 161.3 | 72.8 | 55.9 |
| 17660 | 152.6 | 107.4 | 76.0 |
| 17661 | 148.7 | 104.7 | 67.4 |
| 17662 | 151.2 | 80.9 | 59.6 |
| 17663 | 148.0 | 116.7 | 93.0 |
| 17664 | 214.9 | 158.1 | 129.2 |
| 17665 | 166.1 | 121.4 | 102.5 |
| 17666 | 164.6 | 75.0 | 47.0 |
| 17667 | 153.8 | 105.2 | 100.2 |
| 17668 | 150.8 | 78.6 | 59.5 |
| 17670 | 193.1 | 68.3 | 31.5 |
| 17671 | 159.4 | 81.8 | 63.3 |
| 17672 | 219.6 | 54.2 | 55.2 |
| 17673 | 154.0 | 62.5 | 36.5 |
| 17674 | 253.3 | 73.7 | 46.4 |
| 17675 | 165.0 | 91.3 | 65.1 |
| 17676 | 148.2 | 77.5 | 65.1 |
| 17677 | 151.1 | 102.0 | 78.4 |
| 17678 | 151.1 | 83.6 | 64.7 |
| 17679 | 153.3 | 124.7 | 106.2 |
| 17681 | 167.1 | 68.7 | 51.6 |
| 17682 | 156.4 | 47.2 | 25.4 |
| 17683 | 150.0 | 51.1 | 37.5 |
| 17684 | 170.1 | 85.2 | 57.2 |
| 17685 | 149.2 | 82.4 | 61.5 |
| 17686 | 153.0 | 123.0 | 107.8 |
| 17687 | 145.6 | 83.8 | 57.6 |
| 17688 | 149.5 | 58.4 | 18.2 |
| 17689 | 146.0 | 84.6 | 65.1 |
| 17690 | 119.6 | 68.1 | 45.3 |
| 17691 | 214.8 | 69.2 | 65.2 |
| 17692 | 159.8 | 104.1 | 81.4 |
| 17693 | 146.4 | 112.6 | 74.4 |
| 17694 | 144.3 | 140.3 | 120.2 |
| 17695 | 166.8 | 73.0 | 33.9 |
| 17696 | 153.7 | 75.0 | 53.7 |
| 17697 | 153.4 | 109.1 | 79.4 |
| 17698 | 42.8 | 20.0 | 15.6 |
| 17699 | 154.0 | 54.3 | 31.8 |
| 17700 | 215.3 | 102.8 | 124.6 |
| 17701 | 161.5 | 37.7 | 28.0 |
| 17702 | 142.0 | 92.7 | 62.5 |
| 17703 | 161.3 | 108.5 | 98.3 |
| 17704 | 145.3 | 117.3 | 119.5 |
| 17705 | 231.2 | 106.1 | 84.9 |
| 17706 | 148.3 | 57.8 | 37.6 |
| 17707 | 131.6 | 85.3 | 87.1 |
| 17708 | 150.8 | 64.3 | 56.0 |
| 17709 | 158.6 | 61.9 | 37.3 |
| 17710 | 206.0 | 112.4 | 104.4 |
| 17711 | 181.9 | 100.6 | 73.4 |
| 17713 | 153.7 | 70.0 | 42.7 |
| 17714 | 153.5 | 77.6 | 49.2 |
| 17715 | 150.2 | 64.2 | 56.4 |
| 18725 | 161.8 | 94.7 | 74.8 |
| 18726 | 210.9 | 122.4 | 88.9 |
| 18727 | 153.6 | 78.8 | 72.7 |
| 18730 | 146.7 | 94.2 | 75.3 |
| 18731 | 149.5 | 64.0 | 38.0 |
| 18732 | 172.0 | 43.3 | 22.5 |
| 18733 | 149.4 | 96.5 | 92.7 |
| 18734 | 159.5 | 70.4 | 47.9 |
| 18735 | 164.7 | 96.5 | 85.4 |
| 18736 | 167.8 | 31.1 | 14.2 |
| 18737 | 152.7 | 120.6 | 100.5 |
| 18738 | 147.3 | 52.6 | 30.6 |
| 18739 | 149.2 | 82.6 | 78.3 |
| 18740 | 150.9 | 72.2 | 71.4 |
| 18742 | 151.2 | 148.1 | 127.2 |
| 18743 | 152.3 | 93.1 | 88.3 |
| 18744 | 152.9 | 75.0 | 65.6 |
| 18745 | 54.2 | 32.9 | 27.2 |
| 18746 | 190.4 | 61.8 | 25.7 |
| 18747 | 152.0 | 102.4 | 77.9 |
| 18748 | 145.3 | 91.9 | 77.7 |
| 18749 | 159.7 | 80.2 | 59.2 |
| 18750 | 156.2 | 78.9 | 70.3 |
| 18751 | 150.2 | 64.5 | 45.8 |
| 18752 | 111.7 | 66.5 | 49.3 |
| 18753 | 160.8 | 88.8 | 60.2 |
| 18754 | 145.0 | 66.7 | 34.4 |
| 18755 | 153.7 | 113.6 | 92.7 |
| 18757 | 152.0 | 58.3 | 40.9 |
| 18758 | 124.1 | 118.0 | 103.6 |
| 18759 | 186.6 | 59.7 | 26.9 |
| 18761 | 153.4 | 81.4 | 49.7 |
| 18762 | 152.8 | 95.8 | 69.6 |
| 18763 | 154.1 | 83.4 | 71.4 |
| 18764 | 152.7 | 59.9 | 35.0 |
| 18765 | 148.2 | 104.5 | 111.8 |
| 18766 | 123.0 | 93.2 | 69.2 |
| 18767 | 155.8 | 127.7 | 103.6 |
| 18768 | 175.8 | 68.2 | 36.4 |
| 18769 | 163.7 | 149.3 | 115.2 |
| 18770 | 142.3 | 91.9 | 67.9 |
| 18771 | 158.8 | 47.5 | 25.0 |
| 18772 | 147.1 | 122.5 | 90.4 |
| 18773 | 148.5 | 99.9 | 91.0 |
| 18774 | 139.9 | 73.8 | 56.0 |
| 18775 | 150.2 | 170.8 | 124.7 |
| 18777 | 144.5 | 81.1 | 61.9 |
| 18778 | 158.1 | 86.7 | 59.2 |
| 18779 | 162.6 | 118.6 | 83.4 |
| 18780 | 150.5 | 98.3 | 68.0 |
| 18781 | 142.4 | 82.2 | 40.0 |
| 18782 | 174.4 | 164.4 | 150.2 |
| 18783 | 145.4 | 93.2 | 74.5 |
| 18785 | 142.0 | 81.9 | 61.7 |
| 18786 | 140.1 | 89.6 | 62.7 |
| 18787 | 144.5 | 119.3 | 98.3 |
| 18788 | 147.8 | 78.5 | 50.1 |
| 18789 | 147.1 | 92.5 | 105.7 |
| 18790 | 144.5 | 72.8 | 37.6 |
| 18791 | 151.0 | 129.1 | 130.2 |
| 18792 | 145.4 | 154.5 | 133.8 |
| 18793 | 141.0 | 73.6 | 66.0 |
| 18794 | 44.4 | 13.4 | 10.7 |
| 18796 | 146.5 | 110.4 | 90.2 |
| 18797 | 153.5 | 123.2 | 108.1 |
| 18798 | 150.4 | 60.7 | 26.7 |
| 18799 | 147.2 | 79.8 | 65.5 |
| 18800 | 167.8 | 71.9 | 50.7 |
| 18801 | 122.1 | 68.9 | 50.7 |
| 18802 | 140.6 | 83.3 | 60.8 |
| 18803 | 142.3 | 75.6 | 53.6 |
| 18804 | 134.1 | 46.9 | 37.2 |
| 18805 | 161.0 | 77.9 | 64.8 |
| 18806 | 139.9 | 70.7 | 48.4 |
| 18807 | 147.8 | 76.8 | 65.0 |
| 18808 | 148.3 | 66.5 | 58.8 |
| 18809 | 151.0 | 67.6 | 51.8 |
| 18810 | 141.0 | 77.2 | 51.0 |
| 18811 | 144.9 | 56.9 | 41.0 |
| 18812 | 141.8 | 131.7 | 103.0 |
| 18813 | 196.8 | 160.3 | 136.9 |
| 18814 | 145.7 | 59.9 | 28.4 |
| 18815 | 151.2 | 79.5 | 60.0 |
| 18816 | 146.5 | 56.2 | 40.1 |
| 18817 | 142.5 | 90.4 | 73.0 |
| 18818 | 147.2 | 81.3 | 61.0 |
| 18819 | 147.9 | 73.3 | 58.6 |
| 18820 | 167.5 | 61.4 | 44.8 |
| 18821 | 150.4 | 96.6 | 73.7 |
| 18822 | 26.4 | 18.7 | 14.2 |
| 18824 | 144.3 | 62.5 | 32.4 |
| 18825 | 145.5 | 101.4 | 92.6 |
| 18826 | 149.9 | 56.6 | 31.6 |
| 18827 | 147.4 | 53.2 | 24.6 |
| 18828 | 151.3 | 131.0 | 91.9 |
| 18829 | 147.8 | 55.4 | 43.1 |
| 18830 | 149.7 | 105.2 | 87.8 |
| 18831 | 150.5 | 104.5 | 94.9 |
| 18832 | 144.7 | 114.5 | 103.3 |
| 18833 | 156.6 | 60.1 | 44.3 |
| 18834 | 137.1 | 112.7 | 99.3 |
| 18835 | 149.4 | 74.9 | 60.0 |
| 18836 | 147.3 | 80.7 | 73.4 |
| 18837 | 145.8 | 88.3 | 66.0 |
| 18838 | 148.7 | 56.8 | 39.4 |
| 18839 | 150.7 | 63.5 | 48.1 |
| 21921 | 147.4 | 59.9 | 39.1 |
| 21922 | 155.5 | 130.4 | 111.4 |
| 21923 | 149.8 | 117.5 | 87.2 |
| 21924 | 147.7 | 85.4 | 68.2 |
| 21925 | 150.5 | 58.4 | 55.4 |
| 21926 | 197.7 | 73.5 | 37.9 |
| 21927 | 148.4 | 127.7 | 77.9 |
| 21928 | 151.8 | 68.4 | 109.0 |
| 21929 | 150.0 | 80.6 | 59.1 |
| 21930 | 149.0 | 84.3 | 66.1 |
| 21931 | 146.5 | 79.7 | 53.2 |
| 21932 | 148.1 | 137.3 | 106.3 |
| 21933 | 151.8 | 40.6 | 19.3 |
| 21934 | 153.4 | 56.1 | 39.9 |
| 21935 | 52.0 | 19.2 | 15.8 |
| 21936 | 147.9 | 55.4 | 28.9 |
| 21937 | 149.0 | 65.2 | 39.8 |
| 21938 | 153.5 | 108.1 | 84.7 |
| 21939 | 148.7 | 50.1 | 34.1 |
| 21940 | 26.2 | 25.1 | 22.7 |
| 21941 | 152.7 | 72.6 | 53.2 |
| 21942 | 138.2 | 93.0 | 73.6 |
| 21943 | 151.9 | 44.5 | 33.6 |
| 21944 | 174.7 | 126.4 | 86.1 |
| 21945 | 140.8 | 51.0 | 26.3 |
| 21946 | 158.1 | 61.8 | 16.9 |
| 21947 | 144.1 | 47.5 | 19.8 |
| 21948 | 153.2 | 66.6 | 52.2 |
| 21949 | 164.1 | 46.2 | 21.2 |
| 21950 | 199.6 | 66.4 | 25.9 |
| 21951 | 144.9 | 81.5 | 71.1 |
| 21952 | 151.1 | 54.8 | 49.1 |
| 21953 | 149.3 | 118.3 | 94.3 |
| 21954 | 143.1 | 64.7 | 44.4 |
| 21955 | 148.2 | 72.4 | 62.4 |
| 21956 | 149.1 | 74.6 | 56.3 |
| 21957 | 157.0 | 45.2 | 19.9 |
| 21958 | 141.6 | 70.2 | 52.6 |
| 21959 | 155.7 | 53.0 | 39.4 |
| 21960 | 144.4 | 63.0 | 39.5 |
| 21961 | 145.4 | 86.3 | 67.6 |
| 21962 | 155.3 | 87.9 | 68.5 |
| 21963 | 142.9 | 84.8 | 64.8 |
| 21964 | 141.1 | 82.5 | 73.8 |
| 21965 | 138.8 | 82.1 | 64.8 |
| 21966 | 145.2 | 33.2 | 21.6 |
| 21967 | 147.1 | 43.6 | 27.1 |
| 21968 | 151.0 | 91.9 | 70.7 |
| 21970 | 144.0 | 116.9 | 103.1 |
| 21971 | 148.9 | 107.8 | 85.0 |
| 21972 | 147.7 | 76.0 | 53.7 |
| 21973 | 148.1 | 57.7 | 34.8 |
| 21974 | 163.2 | 19.8 | 9.5 |
| 21975 | 140.8 | 105.6 | 89.2 |
| 21976 | 180.1 | 42.8 | 42.4 |
| 21977 | 149.4 | 91.5 | 92.6 |
| 21978 | 150.0 | 125.9 | 127.8 |
| 21979 | 150.9 | 119.5 | 89.2 |
| 21980 | 146.2 | 108.0 | 92.4 |
| 21981 | 149.5 | 120.5 | 29.3 |
| 21982 | 147.2 | 53.7 | 34.4 |
| 21983 | 150.0 | 86.1 | 68.1 |
| 21984 | 153.9 | 108.1 | 97.0 |
| 21985 | 143.1 | 76.4 | 59.1 |
| 21986 | 151.1 | 63.5 | 46.5 |
| 21987 | 147.9 | 80.5 | 65.9 |
| 21988 | 169.2 | 75.5 | 63.9 |
| 21989 | 164.7 | 45.1 | 13.5 |
| 21990 | 142.1 | 61.1 | 50.2 |
| 21991 | 146.2 | 67.5 | 43.8 |
| 21992 | 145.4 | 59.8 | 39.6 |
| 21993 | 146.9 | 74.4 | 54.0 |
| 21994 | 145.4 | 81.6 | 72.6 |
| 21995 | 134.7 | 122.3 | 114.9 |
| 21996 | 147.5 | 58.9 | 34.2 |
| 21997 | 154.4 | 77.1 | 50.5 |
| 21998 | 135.9 | 61.1 | 48.4 |
| 21999 | 152.0 | 51.2 | 33.5 |
| 22000 | 152.1 | 42.3 | 33.4 |
| 22001 | 147.6 | 73.1 | 64.5 |
| 22002 | 145.4 | 85.7 | 100.3 |
| 22003 | 143.7 | 88.8 | 93.0 |
| 22004 | 150.5 | 102.9 | 86.2 |
| 22005 | 153.4 | 137.2 | 120.4 |
| 22006 | 164.6 | 95.7 | 66.3 |
| 22007 | 148.5 | 57.5 | 38.2 |
| 22008 | 146.3 | 130.9 | 109.2 |
| 22009 | 144.8 | 138.4 | 115.4 |
| 22010 | 140.9 | 107.1 | 94.0 |
| 22011 | 151.6 | 122.7 | 118.0 |
| 22012 | 154.3 | 83.5 | 53.5 |
| 22013 | 151.5 | 107.9 | 82.4 |
| 22014 | 145.0 | 122.5 | 97.8 |
| 22015 | 147.5 | 99.4 | 83.3 |
| 22016 | 145.4 | 123.4 | 90.4 |
| 22017 | 37.3 | 26.5 | 27.8 |
| 22018 | 146.7 | 89.4 | 72.2 |
| 22019 | 147.7 | 104.0 | 84.3 |
| 22020 | 149.5 | 64.3 | 38.6 |
| 22021 | 153.5 | 112.0 | 100.6 |
| 22022 | 142.0 | 92.6 | 65.0 |
| 22024 | 154.6 | 92.7 | 71.1 |
| 22025 | 145.9 | 97.6 | 77.3 |
| 22026 | 93.5 | 43.8 | 30.7 |
| 22027 | 159.3 | 90.3 | 36.0 |
| 22028 | 160.1 | 84.5 | 56.9 |
| 22029 | 147.9 | 108.1 | 74.6 |
| 22030 | 149.4 | 71.6 | 47.7 |
| 22031 | 149.8 | 35.5 | 16.7 |
| 22032 | 150.2 | 101.6 | 102.0 |
| 22033 | 15.2 | 8.95 | 8.17 |
| 22034 | 158.8 | 121.3 | 101.5 |
| 22035 | 143.7 | 51.3 | 23.5 |
| 22037 | 152.4 | 67.6 | 88.0 |
| 22038 | 166.4 | 109.0 | 121.1 |
| 22039 | 48.5 | 12.0 | 6.5 |
| 22616 | 164.7 | 117.4 | 125.8 |
| 22617 | 156.6 | 75.1 | 34.6 |
| 22618 | 141.2 | 95.9 | 64.3 |
| 22619 | 192.5 | 98.9 | 61.9 |
| 22620 | 189.0 | 92.6 | 49.8 |
| 22621 | 157.0 | 96.4 | 73.7 |
| 22622 | 122.5 | 43.9 | 26.3 |
| 22623 | 157.1 | 105.4 | 84.3 |
| 22624 | 142.7 | 131.8 | 97.0 |
| 22625 | 162.4 | 104.7 | 70.8 |
| 22626 | 155.7 | 94.0 | 60.6 |
| 22627 | 176.0 | 108.1 | 85.9 |
| 22628 | 158.3 | 98.3 | 69.5 |
| 22629 | 154.0 | 82.5 | 53.8 |
| 22630 | 170.8 | 70.1 | 49.7 |
| 22631 | 159.6 | 108.9 | 70.4 |
| 22632 | 155.5 | 127.4 | 97.4 |
| 22634 | 158.9 | 89.9 | 82.8 |
| 22636 | 160.5 | 54.8 | 38.4 |
| 22637 | 161.3 | 67.5 | 45.4 |
| 22638 | 114.9 | 95.08 | 86.31 |
